# Single-Access Transfemoral TAVI with a Balloon Expandable Valve: Age-Stratified Analysis of Clinical Outcomes

**DOI:** 10.3390/jcm15166305

**Published:** 2026-08-14

**Authors:** Sushant Saluja, Muntaser Omari, Omran Abukhalaf, Debbie Stewart, Sarah Lamb, Timothy Cartlidge, Richard Edwards, Rajiv Das, Azfar Zaman, Mohamed Farag, Mohammad Alkhalil

**Affiliations:** 1 Division of Cardiovascular Sciences, Faculty of Biology, Medicine and Health, University of Manchester, Manchester M13 9PL, UK; 2 Cardiothoracic Centre, Freeman Hospital, Newcastle-upon-Tyne NE7 7DN, UK; 3 Translational and Clinical Research Institute, Newcastle University, Newcastle-upon-Tyne NE2 4AE, UK; 4 Department of Cardiothoracic Services, Freeman Hospital, Freeman Road, Newcastle-upon-Tyne NE7 7DN, UK

**Keywords:** transcatheter aortic valve implantation, single access, dual access, age, VARC-3, MACE

## Abstract

**Background**: Single-access transfemoral transcatheter aortic valve implantation (TAVI) reduces the need for multiple vascular access and therefore, the related risk of related complications. However, the impact of patient age on its clinical outcomes remains uncertain. **Methods:** This is a secondary analysis of previously reported propensity score matched study comparing clinical outcomes of single- versus dual-access transfemoral TAVI using SAPIEN-3-Ultra. We examined the relation between patient age, access strategy and outcomes—specifically, 30-day safety endpoint according to the Valve Academic Research Consortium 3 (VARC-3) and major adverse cardiovascular events (MACE), defined as death, permanent pacemaker, neurological or vascular complications—using logistic regression interaction analyses, and restricted cubic spline modelling. **Results:** A total of 204 patients (propensity score-matched cohort) were included in the study. The elderly group were more likely to have hypertension, peripheral vascular disease or stroke. The use of single access was comparable. The elderly cohort demonstrated a tendency toward longer fluoroscopy and procedural times compared with the non-elderly group. This pattern likely reflects the greater technical complexity and heightened procedural caution often associated with older patients, including more challenging vascular anatomy and the need for careful device handling. Although not statistically significant, these findings remain clinically meaningful and are consistent with the notion that procedures in elderly patients frequently require more meticulous planning and execution. There was a significant interaction between access strategy and age in reducing MACE, but not in VARC-3 safety outcomes. Single access was associated with a significant reduction in MACE (5.4% vs. 26.7%, *p* = 0.015) with comparable technical and safety outcomes when compared with elderly patients who underwent dual-access TAVI. **Conclusions:** Single-access TAVI was linked to favourable early outcomes, maintained procedural success, and suggested a possible age-dependent pattern in the occurrence of MACE. Our findings support the need for randomised studies to demonstrate superiority of single- versus dual-access TAVI, particularly in the elderly population.

## 1. Introduction

Transcatheter aortic valve implantation (TAVI) has revolutionised the therapeutic approach for severe symptomatic aortic stenosis, establishing itself as a standard of care across various risk categories [1,2,3]. This was supported by landmark randomised trials demonstrating noninferiority and, often, superiority compared with surgical aortic valve replacement [4,5]. Nonetheless, procedural complications, including vascular, neurological and permanent pacemaker, remain a challenge in patients undergoing transfemoral TAVI. Such complications are associated with higher mortality, prolonged hospitalisation, and increased healthcare costs [6,7].

Single-access TAVI has emerged as a minimalist approach to reduce procedural complications [8,9]. It was associated with reductions in procedural time, contrast use, and radiation exposure [8,9]. Additionally, it had lower vascular complications and permanent pacemaker rates compared to dual-access TAVI [8]. Single-access TAVI involves performing all procedural steps through a single arterial access, thereby eliminating the need for a second access during valve deployment. The positioning of the transcatheter heart valve is guided by calcified landmarks, predominantly using the aortic valve leaflets, resulting in higher implants which have been associated in observational studies with lower rates of permanent pacemaker implantation [8,9].

TAVI procedural complications increase with age [8,10,11]. In a large dataset of almost 85,000 patients, octogenarians who underwent TAVI had a higher rate of stroke and permanent pacemaker compared to younger patients [12]. This was associated with 40% increase in mortality in this age group [12]. Whether the minimalist approach using single-access TAVI would affect clinical outcomes in elderly patients remains to be determined. The parent propensity-matched study demonstrated procedural and early safety advantages of single-access TAVI over dual-access TAVI [8]. However, whether these effects are consistent across age groups remains unknown. Given the established association between advanced age and procedural risk after TAVI, we therefore performed a secondary age-stratified analysis to determine whether age modifies the relationship between access strategy and early clinical outcomes. In the present study, we defined elderly patients as those aged ≥84 years, as this represented the oldest tertile of our cohort and provided a pragmatic balance between clinical relevance and subgroup size for exploratory analysis. A biological interaction between age and access strategy may be plausible, as older patients often exhibit more pronounced vascular calcification, arterial tortuosity, and frailty, which may amplify the procedural burden of additional vascular access and catheter manipulation [13]. Consequently, the relative advantage of a single-access strategy may differ according to age. The aim of this analysis was to examine the relation between age, access strategy (single versus dual), and early clinical outcomes in patients who underwent TAVI with balloon-expandable SAPIEN 3 Ultra.

## 2. Methods

This is a secondary analysis utilising a propensity-matched cohort of recently reported data on the safety of single- versus dual-access TAVI [8]. In brief, consecutive patients with severe aortic stenosis who underwent a transfemoral TAVI procedure with a balloon-expandable valve (SAPIEN 3 Ultra, Edwards Lifesciences LLC, Irvine, CA, USA) between January 2022 and August 2024 at a single UK centre were evaluated. Data were prospectively collated, maintained, and regularly updated by data managers to ensure accuracy and data completeness. The study was exempted from an institutional board review and the formal ethics process as it was part of a clinical audit.

All patients had severe native aortic stenosis or degeneration of a surgical bioprosthetic valve. The severity of aortic valve disease was defined according to current guidelines and following discussion at the local multidisciplinary team meeting [1]. Patients with low-gradient severe aortic stenosis and both classical or paradoxical low flow were included if they met the required criteria to secure the diagnosis of severe aortic stenosis using aortic valve calcium scoring or dobutamine stress echocardiography [1]. Exclusion criteria included patients who underwent a non-transfemoral approach, used self-expanding valve platforms, or received a non-SAPIEN balloon-expandable valve. MDCT was performed as part of procedural planning and images were analysed using 3mensio software version 9.2 (3mensio Structural Heart, 3mensio Medical Imaging, Maastricht, The Netherlands) [14,15].

**Figure 1:**
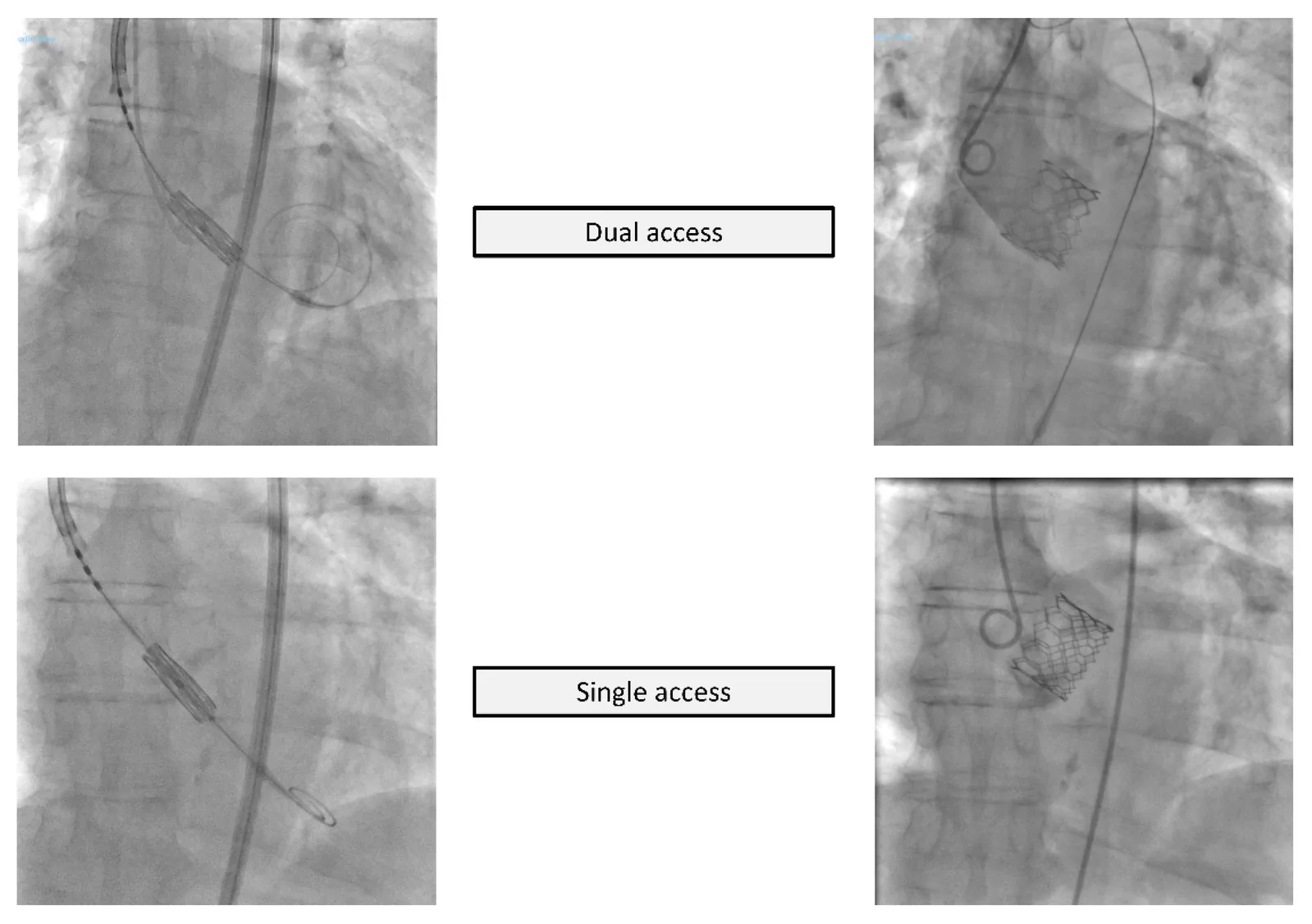
Illustration of single- versus dual-access TAVI procedures.

All cases were performed under local anaesthesia and conscious sedation. A single-access procedure has been previously described [8]. In summary, an E-Sheath was inserted as per standard practice following deployment of closure devices (Perclose ProGlide, Abbott Vascular, Santa Clara, CA, USA). An aortogram was performed using a pigtail catheter advanced through the E-Sheath, and the resulting image served as a procedural roadmap to guide valve deployment. After this point, every effort was made to not move the table or image intensifier. The dual-access TAVI was performed according to standard practice, with a secondary access obtained via the contralateral femoral or radial artery for the pigtail catheter (Figure 1). The decision regarding pre-dilation or post-dilation was according to the operator’s discretion.

The clinical endpoints were technical success and 30-day safety, as defined by the Valve Academic Research Consortium 3 (VARC-3) [16]. Technical success was defined as successful delivery of the transcatheter heart valve into the correct position without procedural death, or surgical intervention related to the device, or to a major vascular or access-related, or cardiac structural complication. The 30-day safety endpoint was defined as freedom from all-cause mortality, all stroke, VARC type 2–4 bleeding, major vascular, access-related, or cardiac structural complications, acute kidney injury stage 3 or 4, significant aortic regurgitation, new permanent pacemaker, or freedom from surgical or intervention related to the device. Additionally, we assessed the relationship between access strategy and major adverse cardiovascular events (MACE) at 30 days, defined as the composite of death, new permanent pacemaker, neurological or vascular complications. These endpoints were defined according to the VARC-3 criteria [16]. For neurological events, this included ischaemic and haemorrhagic strokes, symptomatic hypoxic-ischaemic injury (defined as neurological dysfunction secondary to hypotension or hypoxia), or transient ischemic attack (TIA). Transient delirium post-procedure without evidence of neurological injury on imaging was not classified as a neurological event and not included in the definition of MACE. For vascular complications, both major and minor complications, defined according to the VARC-3 criteria, were included in the MACE definition.

This study examined an age-focused analysis of 30-day safety and MACE. Previous studies highlighted an increased procedural risk and mortality in patients undergoing TAVI with advanced age [17,18]. Using age-stratified cut-off, previous studies highlighted that patients above age 84 had worse clinical outcomes compared to younger population [19]. This cut-off is comparable to the oldest tertile within our recruited patients (≥ 84 years). Collectively and for the purpose of the current analysis, we defined elderly patients as those who underwent TAVI and are 84 years old or above. This cut-off corresponded to the oldest tertile of the study cohort and was selected to provide a pragmatic balance between clinical relevance and sufficient sample size for exploratory age-stratified analyses.

We evaluated the role of single-access TAVI, compared with dual-access TAVI, on 30-day safety and MACE endpoints in elderly (≥84) and non-elderly (<84) groups.

### Statistical Analysis

Analyses were performed using Python version 3.11 [20] and IBM SPSS Statistics version 31 (IBM Corp., Armonk, NY, USA) [21]. Descriptive statistics and categorical comparisons (including chi-square tests for age cut-offs) were performed in SPSS, while regression and spline analyses were conducted in Python. The analytic dataset included 204 patients with complete data for study identifier, age, access strategy, safety, and MACE.

Continuous variables were presented as mean ± standard deviation or median ± interquartile range, as appropriate, and were compared using the unpaired t-test or the Wilcoxon rank-sum test. Categorical variables were compared using the chi-square test or Fisher’s exact test, as appropriate.

Logistic regression was used to estimate odds ratios with 95% confidence intervals and Wald *p*-values. Three models were fitted for each outcome: a crude model including only access strategy; an age-adjusted model including access strategy and age; and an interaction model including access strategy, age, and the access-by-age interaction term. Linearity of the age effect was assessed using the Box-Tidwell method. Across the cohort, there were 43 Safety events, 29 Wide MACE, and 21 Narrow MACE. For the age-adjusted logistic regression models, the approximate events-per-variable ratio was 21.5 for Safety, 14.5 for Wide MACE, and 10.5 for Narrow MACE. In the models including the access-by-age interaction term, the corresponding ratios were 14.3, 9.7, and 7.0, respectively.

To assess potential non-linear associations, restricted cubic spline models were fitted with 3 degrees of freedom and 4 knots placed at the 5th, 35th, 65th, and 95th percentiles of age. Models were centred at the median age of 80.5 years. Point estimates and 95% confidence intervals were plotted across the observed age range.

Effect modification was assessed using the *p*-value for the access-by-age interaction term, and the interaction was explored descriptively across age strata defined by tertiles. Sensitivity analyses additionally adjusted for the propensity variables used in the parent study, including sex, body mass index, prior percutaneous coronary intervention, previous stroke or transient ischaemic attack, peripheral vascular disease, frailty, valve size, and left ventricular systolic function.

All tests were two-sided, and *p*-values < 0.05 were considered statistically significant. No adjustment for multiple comparisons was made because of the exploratory nature of the analysis. As an exploratory analysis, we additionally performed chi-square tests to compare 30-day MACE rates using a dichotomous age cut-off of ≥84 years.

## 3. Results

A total of 204 patients (propensity score matched cohort) were included in the study; 102 in the single-access group and 102 in the dual-access group. There were 67 patients in the elderly group and 137 patients in the non-elderly group. The elderly group were more likely to have hypertension (72% vs. 56%, *p* = 0.026), peripheral vascular disease (24% vs. 10%, *p* = 0.01) or stroke (12% vs. 4%, *p* = 0.023). They were less likely to be diabetic (19% vs. 34%, *p* = 0.036) and had lower body mass index (26 ± 4 vs. 28 ± 6, *p* = 0.06) compared to the non-elderly group. Baseline clinical characteristics stratified by age are shown in Table 1.

The use of single access was comparable among the elderly and non-elderly group (55% vs. 47%, *p* = 0.30). Among patients who underwent TAVI with dual access, the secondary access was radial in 87% in the whole cohort without statistical difference among patients in the elderly and non-elderly groups (Table 2). The use of pre-dilation and post-dilation was similar between the two groups, but larger valve size was more frequently used in the non-elderly group [26 (26–29) vs. 26 (26–26), *p* = 0.013]. Notably, the elderly group had longer fluoroscopy time (10.9 ± 7.3 vs. 9.1 ± 4.9, *p* = 0.065) and procedural time (66 ± 30 vs. 59 ± 24, *p* = 0.078) compared to the non-elderly group.

There were no differences in clinical outcomes, including technical success, safety, MACE, and its individual components among elderly and non-elderly patients, with a higher trend towards neurological events, and bleeding and vascular complications in the elderly group (Table 3).

When the data were analysed according to access strategy, single access was associated with better odds of safety [odds ratio (OR) 0.46, (95% CI 0.23 to 0.92), *p* = 0.028] and MACE [OR 0.40, (95% CI 0.17 to 0.92), *p* = 0.031] compared to dual access in the overall cohort. These associations remained similar after adjustment for age.

**Figure 2:**
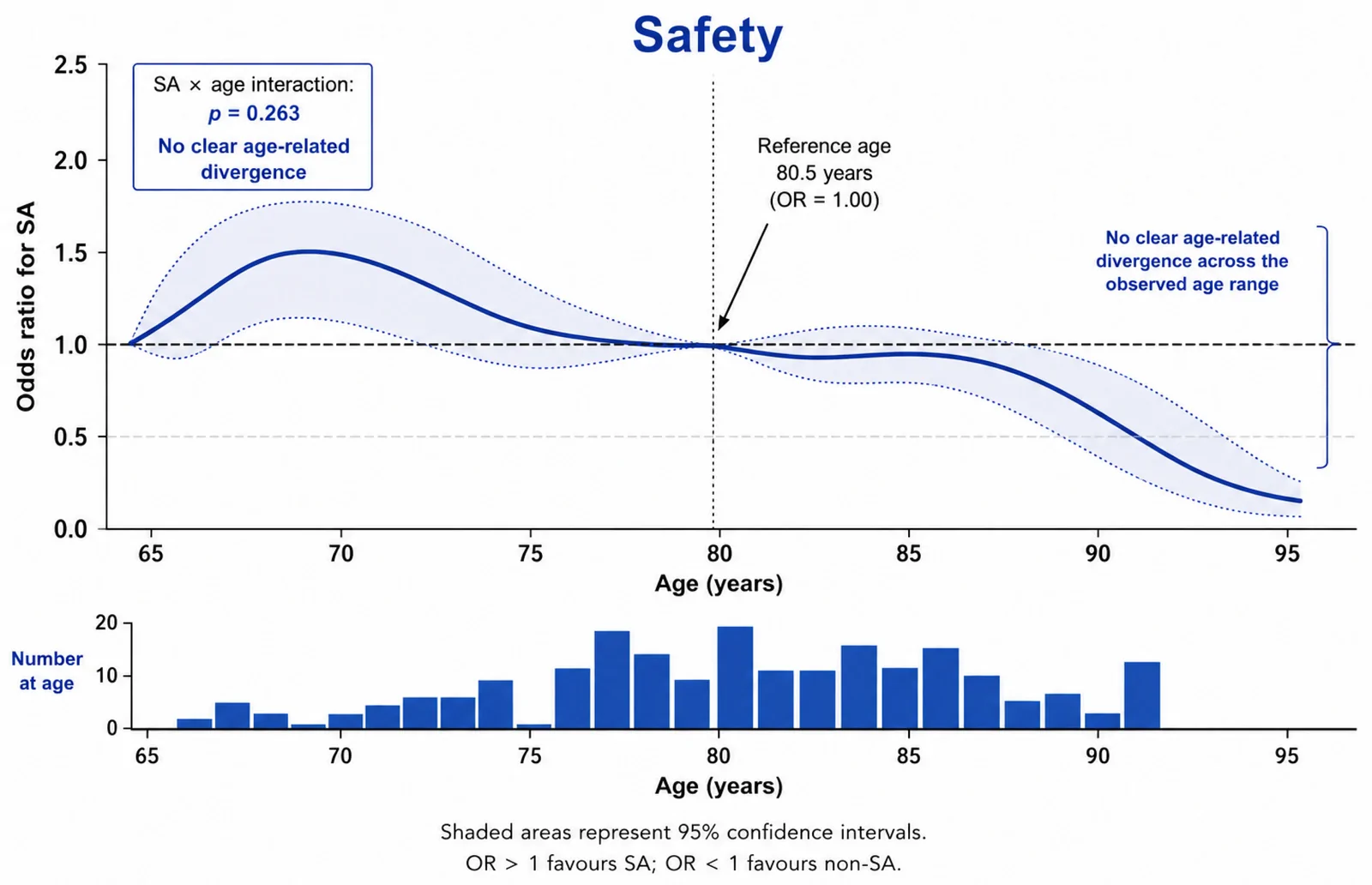
Restricted cubic spline analysis for safety. Restricted cubic spline curve showing the age-dependent association between single-access and the VARC-3 30-day Safety endpoint. The reference age was set to the cohort median of 80.5 years, with the odds ratio centred at 1.0. The solid line represents the estimated odds ratio across age, and the shaded band represents the 95% confidence interval. The lower panel displays the distribution of observations across the age spectrum. There was no clear evidence of age-related divergence in the association between single-access and Safety (interaction *p* = 0.263), although the confidence intervals widened at the extremes of the age range where fewer observations were available.

**Figure 3:**
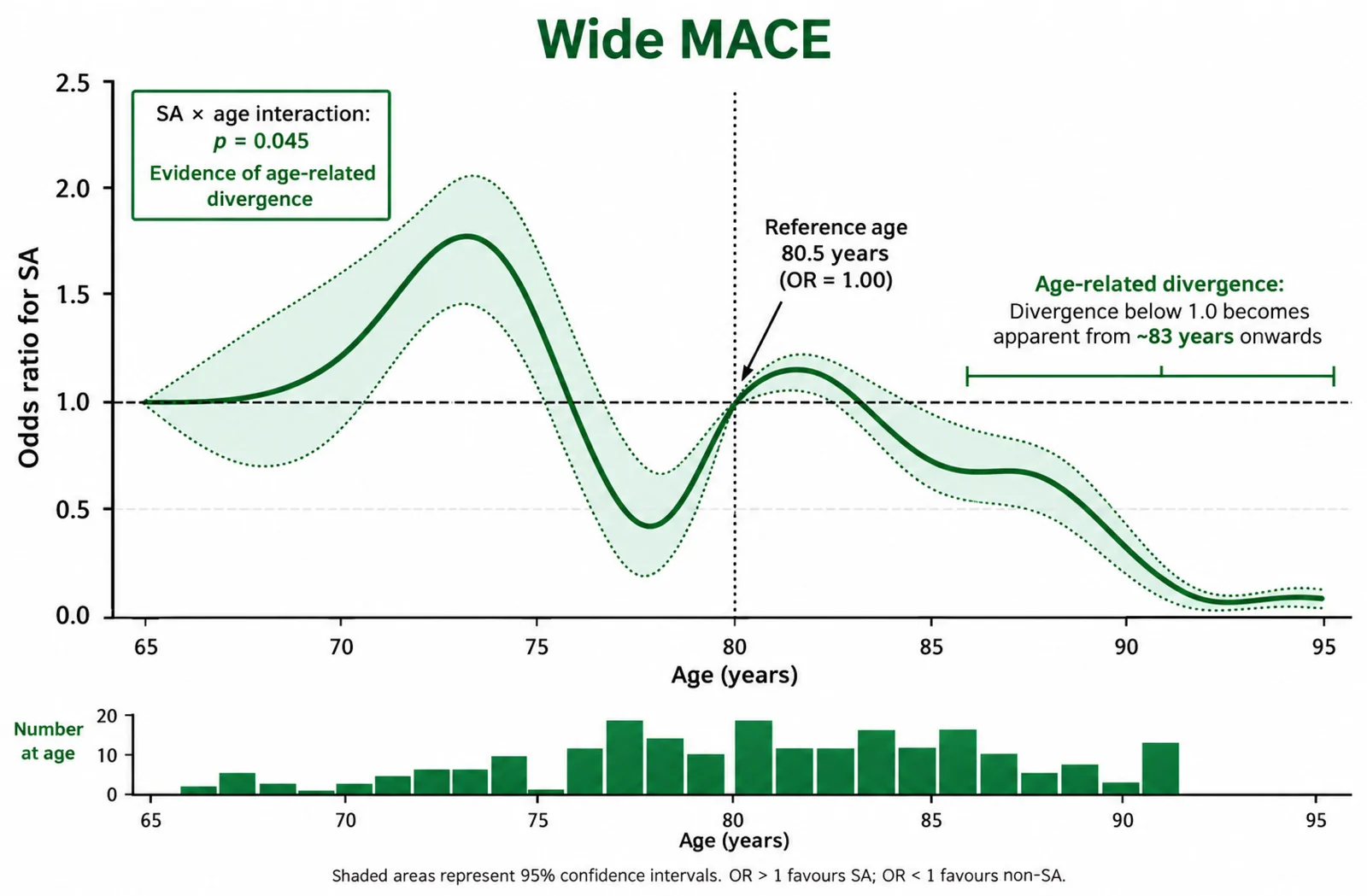
Restricted cubic spline analysis for MACE. Restricted cubic spline curve showing the age-dependent association between single-access and 30-day Wide MACE. The reference age was set to the cohort median of 80.5 years, with the odds ratio centred at 1.0. The solid line represents the estimated odds ratio across age, and the shaded band represents the 95% confidence interval. The lower panel displays the distribution of observations across the age spectrum. There was evidence of age-related divergence in the association between single-access and Wide MACE (interaction *p* = 0.045), with greater separation between the estimated odds ratios at older ages. Confidence intervals widened at the extremes of the age range where fewer observations were available.

Age was not independently associated with safety [OR 0.99, (95% CI 0.95 to 1.04), *p* = 0.68] or MACE [OR 1.03, (95% CI 0.97 to 1.09), *p* = 0.37], suggesting a lack of a simple linear relationship with the studied outcomes. The access-by-age interaction was not significant for safety, but it was significant for MACE. The interaction *p*-values were 0.263 for safety and 0.045 for MACE; however, the confidence intervals were wide, and the elderly subgroup was small, so this finding should be interpreted cautiously. The restricted cubic spline plots showed little evidence of meaningful age-related modification for safety, whereas the MACE endpoints demonstrated a more pronounced age-dependent pattern, particularly at older ages (Figure 2 and Figure 3). Although a significant interaction between age and access strategy was observed for MACE, this finding should be interpreted cautiously, given the relatively small number of patients aged ≥84 years and the wide confidence intervals around the estimates and should therefore be considered exploratory and hypothesis-generating.

Single access was associated with a significant reduction in MACE in the elderly group (5.4% vs. 26.7%, *p* = 0.015) with similar technical (2.7% vs. 6.7%, *p* = 0.44) and safety outcomes (10.8% vs. 20.0%, *p* = 0.29) when compared with elderly patients who underwent dual-access TAVI (Table 4). The reduction in MACE was driven predominantly by fewer neurological and vascular events in the single-access elderly group, whereas mortality and permanent pacemaker implantation were infrequent and contributed less to the overall difference. There were no differences between single- and dual-access strategies in technical success, safety, or MACE in the non-elderly group (Table 4).

## 4. Discussion

The main findings from this study are as follows: firstly, elderly patients undergoing TAVI procedures had more co-morbidities, longer fluoroscopy and procedural times but comparable use of single access to the non-elderly group; secondly, age and access strategy had a non-linear relationship with significant interaction and reductions in MACE in elderly patients who underwent single-access TAVI; and thirdly, age did not modify the role of single-access TAVI regarding 30-safety outcomes.

Different strategies have been proposed to reduce procedural complications during TAVI with varying success rates. This includes the use of the cusp-overlap technique to decrease pacemaker rate, cerebral protection device to minimise stroke risk, and a combined suture/plug strategy to manage vascular access closure [22,23,24]. Single access has emerged as a new strategy to streamline the TAVI procedure (when using a balloon-expandable valve with Edwards Sapien) and was recently shown to be associated with a significant reduction in pacemaker rate and paravalvular regurgitation [8].

Previous studies have highlighted the increased procedural risks with advance age [17,18]. Although our study highlighted comparable safety outcomes, there was a trend towards higher neurological and vascular complications as well as bleeding events in the elderly compared to the non-elderly group. The higher prevalence of hypertension, peripheral vascular disease, prior stroke, and diabetes in the elderly group likely reflects a greater burden of systemic atherosclerosis and frailty, which may have contributed to procedural complexity and early clinical events [25]. Accordingly, the observed age-related differences should be interpreted as reflecting both chronological age and associated comorbidity burden rather than age alone. Additionally, fluoroscopic and procedural timing were longer in the former group, and likely to reflect anatomical challenges commonly seen in this age group. Therefore, the age-stratified analysis offers additional clinical insights given the differential observed procedural risks in our cohort. While age did not significantly alter the relation between access strategy and safety outcomes, evidence suggests that age impacted the association with adverse clinical events, namely death, neurological events, vascular complications and new pacemaker. Because MACE combines events of varying clinical severity, the observed reduction should be interpreted in the context of its component events. In this cohort, the main signal appeared to relate to fewer access-related and neurological complications rather than to mortality or pacemaker implantation, although the small number of events limits definitive component-wise inference. This trend was more pronounced in the elderly group, but it was evident in those over 80, indicating the advantages of minimising arterial access in the very elderly patients. This should not be surprising given that vascular vulnerability, frailty, and procedural complexity escalate with age.

Taken together, these findings expand the current understanding of minimalist transfemoral TAVI strategies. Previous observational studies, propensity-matched analyses, and pooled meta-analyses have shown that reducing or eliminating secondary arterial access can decrease contrast exposure, fluoroscopy time, radiation dose, and access-related complications without compromising procedural success [8,26]. This study further reinforces this evidence by demonstrating that, in a modern balloon-expandable TAVI cohort, single-access not only preserves these procedural benefits but also appears to offer a favourable early safety profile across age groups, with potential clinical significance in older patients.

The broader clinical implication is that single access appears to be a safe and feasible, less invasive option for transfemoral TAVI rather than merely a technical variation. Its appeal lies in the combination of procedural simplicity, reduced operator and patient radiation exposure, and safety of the TAVI procedure. Simultaneously, the age-related findings emphasise the importance of individualised procedural planning. As the TAVI population continues to expand to include both younger, lower-risk patients and increasingly frail older patients, understanding how access strategies interact with age will be essential for optimising procedural selection and enhancing outcomes.

Additional vascular access is inherently associated with bleeding and vascular complications. Recent contemporary data highlighted that this risk is 0.5–1.0%, even when using the radial artery [27]. Importantly, this data had much lower average age and included only 10% of patients above the age of 80. In comparison, our study cohort had almost half of its patients above the age of 80. Therefore, factors such as iliofemoral or brachiocephalic tortuosity, calcification and frailty need to be taken into consideration as they may increase procedural complications. Moreover, additional interaction between catheters and vascular wall can be associated with increased stroke risk, particularly when navigating tortuous brachiocephalic system in the elderly population. This was reported in a study comparing upper-body versus lower-body access for endovascular aortic repair [28].

The increased adoption of single access depends on addressing questions about patient selection, particularly among elderly individuals. Our findings are hypothesis-generating and indicate that older patients may experience a greater relative benefit from a single arterial technique. However, this observation is based on a non-randomised secondary analysis and warrants confirmation through prospective studies specifically designed to evaluate this interaction.

The notable interaction between age and access strategy for MACE indicates that the potential advantage of single-access TAVI may be more evident in older patient populations. This can plausibly be attributed to the increased vulnerability of elderly patients to the cumulative procedural burdens associated with dual access, such as additional vascular punctures, manipulation, and prolonged procedural duration, all of which may elevate the risk of adverse cardiovascular events [29]. Conversely, the broader VARC-3 safety endpoint encompasses complications that are likely influenced more by baseline patient characteristics or device-related factors, which may account for the lack of a similar interaction. The analysis employing restricted cubic spline modelling corroborates a continuous, age-related modification of treatment effect, rather than a strict threshold, thereby indicating that age should be regarded as a modifier of effect when evaluating access strategy.

From an academic perspective, current ESC/EACTS guidelines endorse a customised procedural approach informed by anatomical and clinical factors, with a growing preference for minimalist techniques when appropriate [30]. In this framework, our results indicate that single-access TAVI may be particularly advantageous for elderly patients; however, these findings are not sufficient to alter standard patient selection criteria or clinical protocols. Instead, they should be regarded as hypothesis-generating evidence warranting further investigation through prospective randomised trials before endorsing broader implementation.

Our study has limitations: it was a secondary analysis of a propensity score-matched cohort from a single centre with over 6 years of experience with a single-access approach. The data were not randomised, and the decision to use single access was left to the operator’s discretion. Finally, the results are applicable only to balloon-expandable platforms from Edwards Lifesciences and cannot be extrapolated to self-expanding or other balloon-expandable platforms. Another significant limitation of this analysis is the relatively small size of the elderly subgroup, which diminishes statistical power and elevates the likelihood of a type II error. Consequently, some clinically meaningful numerical differences that fail to achieve statistical significance should be interpreted with caution and not regarded as evidence of equivalence between access strategies. Accordingly, the findings should be viewed as preliminary and supportive of further prospective studies rather than conclusive proof of superiority.

Furthermore, despite propensity score matching, residual confounding may persist due to operators’ discretionary choice of a single- or dual-access strategy. Unmeasured procedural and anatomical variables, such as iliofemoral calcification, vessel tortuosity, vascular calibre, operator expertise, and temporal learning effects, could have influenced both access modality and clinical outcomes. Additionally, these findings stem from a single high-volume centre with extensive experience in single-access TAVI procedures and are specific to the balloon-expandable SAPIEN 3 Ultra platform. Consequently, the generalizability of these results to self-expanding valves, other balloon-expandable systems, or centres with differing procedural volumes, operator proficiency, or access strategies is limited. An additional limitation is the relatively low number of adverse events compared with the number of variables incorporated into the interaction and spline models, which may predispose to overfitting. Therefore, these analyses should be regarded as exploratory and hypothesis-generating rather than as definitive.

In conclusion, our findings indicate that single-access TAVI may offer advantages for elderly patients. However, these results should be regarded as preliminary and require validation through larger, prospective studies. Our data underscore the necessity for randomized trials to compare the efficacy of single- versus dual-access TAVI, especially in the geriatric population.

## Figures and Tables

**Table 1:** Baseline clinical and echocardiographic characteristics of included patients, stratified according to age.

	Whole Cohort *n* = 204	Non-Elderly *n* = 137	Elderly *n* = 67	*p* Value
Age (mean ± SD)	80 ± 8	76 ± 7	87 ± 3	<0.001
Male gender (*n*, %)	169 (83%)	112 (82%)	57 (85%)	0.55
Body mass index (mean ± SD)	27 ± 5	28 ± 6	26 ± 4	0.06
Elective admission (*n*, %)	146 (72%)	100 (73%)	46 (69%)	0.52
Diabetes (*n*, %)	59 (29%)	46 (34%)	13 (19%)	0.036
Hypertension (*n*, %)	124 (61%)	76 (56%)	48 (72%)	0.026
Previous smoker (*n*, %)	91 (45%%)	59 (43%)	32 (48%)	0.53
Obstructive lung disease (*n*, %)	35 (17%)	24 (18%)	11 (16%)	0.85
Severe liver disease (*n*, %)	5 (3%)	5 (4%)	0 (0%)	0.11
Creatinine (mean ± SD)	120 ± 97	126 ± 114	109 ± 45	0.23
Haemoglobin (mean ± SD)	128 ± 21	127 ± 21	128 ± 22	0.88
Coronary artery disease (*n*, %)	36 (31%)	21 (26%)	15 (40%)	0.15
Previous PCI (*n*, %)	21 (10%)	12 (9%)	9 (13%)	0.30
Peripheral vascular disease (*n*, %)	30 (15%)	14 (10%)	16 (24%)	0.01
Previous CVA/TIA (*n*, %)	13 (6%)	5 (4%)	8 (12%)	0.023
Previous CABG (*n*, %)	29 (14%)	21 (15%)	8 (12%)	0.52
Atrial fibrillation (*n*, %)	57 (28%)	36 (26%)	21 (31%)	0.45
NYHA III/IV (*n*, %)	159 (78%)	111 (81%)	48 (72%)	0.13
Frail (*n*, %)	17 (8%)	10 (7%)	7 (10%)	0.45
Mean gradient (mean ± SD)	45 ± 13	46 ± 14	43 ± 13	0.29
Aortic valve area (mean ± SD)	0.77 ± 0.69	0.80 ± 0.83	0.71 ± 0.18	0.44
Bicuspid morphology (*n*, %)	3 (2%)	1 (1%)	2 (3%)	0.21
Valve-in-valve (*n*, %)	7 (3%)	7 (5%)	0 (0%)	0.06
Preserved LV function (*n*, %)	139 (68%)	90 (66%)	49 (73%)	0.28

CABG: Coronary artery bypass graft; CVA: Cerebrovascular event; LV: Left ventricle; NYHA: New York Heart Association; PCI: Percutaneous coronary intervention; SD: Standard deviation; TIA: Transient ischemia attack.

**Table 2:** Procedural characteristics of included patients, stratified according to age.

	Whole Cohort *n* = 204	Non-Elderly *n* = 137	Elderly *n* = 67	*p* Value
Single access (*n*, %)	102 (50%)	65 (47%)	37 (55%)	0.30
Main access (right femoral) (*n*, %)	168 (83%)	113 (84%)	55 (82%)	0.77
Secondary access (femoral) (*n*, %)	13 (13%)	10 (14%)	3 (10%)	0.59
Pre-dilation (*n*, %)	29 (14%)	16 (12%)	13 (19%)	0.14
Pre-dilation balloon size (median, IQR)	20 (18–21)	20 (18–23)	18 (18–20)	0.11
Valve size (median, IQR)	26 (26–29)	26 (26–29)	26 (26–26)	0.013
Post-dilation (*n*, %)	24 (12%)	13 (10%)	11 (17%)	0.14
Post-dilation balloon size (median, IQR)	26 (24–29)	26 (26–29)	26 (23–29)	0.39
Contrast use (mean ±SD)	60 ± 42	57 ± 40	65 ± 47	0.24
Fluoroscopy time (mean ± SD)	9.7 ± 5.9	9.1 ± 4.9	10.9 ± 7.3	0.065
Radiation dose (DAP) (mean ± SD)	221 ± 150	237 ± 166	192 ± 108	0.066
Procedural duration (mean ±SD)	62 ± 26	59 ± 24	66 ± 30	0.078

DAP: Dose area product; IQR: Interquartile range, SD: Standard deviation.

**Table 3:** Clinical outcomes of included patients, stratified according to age.

	Whole Cohort *n* = 204	Non-Elderly *n* = 137	Elderly *n* = 67	*p* Value
Technical success * (*n*, %)	8 (3.9%)	5 (3.6%)	3 (4.5%)	0.78
30-day safety ⁋ (*n*, %)	43 (21.1%)	33 (24.1%)	10 (14.9%)	0.13
Major adverse cardiovascular events ⁋⁋ (*n*, %)	29 (14.2%)	19 (13.9%)	10 (14.9%)	0.84
Death at 30 days (*n*, %)	3 (1.5%)	3 (2.2%)	0 (0%)	0.22
Major vascular complication (*n*, %)	3 (1.5%)	2 (1.5%)	1 (1.5%)	0.99
Any vascular complications (*n*, %)	7 (3.4%)	3 (2.2%)	4 (6.0%)	0.16
Neurological events (*n*, %)	7 (3.4%)	3 (2.2%)	4 (6.0%)	0.16
Stroke (*n*, %)	3 (1.5%)	2 (1.5%)	1 (1.5%)	0.99
Any bleeding events (*n*, %)	9 (4.4%)	4 (2.9%)	5 (7.5%)	0.14
Severe PVL (*n*, %)	4 (2.0%)	4 (2.9%)	0 (0%)	0.16
New pacemaker (*n*, %)	14 (6.9%)	11 (8.0%)	3 (4.5%)	0.35
Mean gradient ≥ 20 mmHg (*n*, %)	13 (8.0%)	9 (8.1%)	4 (7.8%)	0.95

* Defined technical success at the exit from the procedural room according to the Valve Academic Research Consortium 3 (VARC-3). This was defined as freedom from mortality and surgical intervention related to the transcatheter heart valve, major vascular, access site or cardiac complications. ⁋ This was defined using VARC-3 early safety at 30 days as freedom from all-cause mortality, all stroke, VARC type 2–4 bleeding, major vascular, access-related, or cardiac structural complication, acute kidney injury stage 3 or 4, severe aortic regurgitation, or new permanent pacemaker. ⁋⁋ This was defined as the composite of death, new permanent pacemaker, neurological or vascular complications at 30 days.

**Table 4:** Clinical outcomes of included patients, according to vascular access strategy.

	Non-Elderly Group *n* = 137			Elderly Group *n* = 67		
	Single Access *n* = 65	Dual Access *n* = 72	*p* Value	Single Access *n* = 37	Dual Access *n* = 30	*p* Value
Technical success * (*n*, %)	3 (4.6%)	2 (2.8%)	0.57	1 (2.7%)	2 (6.7%)	0.44
30-day safety ⁋ (*n*, %)	11 (16.9%)	22 (30.6%)	0.06	4 (10.8%)	6 (20.0%)	0.29
Major adverse cardiovascular events ⁋⁋ (*n*, %)	7 (10.8%)	12 (16.7%)	0.32	2 (5.4%)	8 (26.7%)	0.015

* Defined technical success at the exit from the procedural room according to the Valve Academic Research Consortium 3 (VARC-3). This was defined as freedom from mortality and surgical intervention related to the transcatheter heart valve, major vascular, access site or cardiac complications. ⁋ This was defined using VARC-3 early safety at 30 days as freedom from all-cause mortality, all stroke, VARC type 2–4 bleeding, major vascular, access-related, or cardiac structural complication, acute kidney injury stage 3 or 4, severe aortic regurgitation, or new permanent pacemaker. ⁋⁋ This was defined as the composite of death, new permanent pacemaker, neurological or vascular complications at 30 days.

## Data Availability

The original contributions presented in this study are included in the article. Further inquiries can be directed to the corresponding author.
